# ADP-ribosylation factor 1 expression regulates epithelial-mesenchymal transition and predicts poor clinical outcome in triple-negative breast cancer

**DOI:** 10.18632/oncotarget.7515

**Published:** 2016-02-18

**Authors:** Sabrina Schlienger, Shirley Campbell, Sarah Pasquin, Louis Gaboury, Audrey Claing

**Affiliations:** ^1^ Department of Pharmacology, Faculty of Medicine, Université de Montréal, Montreal, Canada; ^2^ Department of Pathology and Cell Biology, Institute for Research in Immunology and Cancer, Université de Montréal, Montreal, Canada

**Keywords:** ADP-ribosylation factor (ARF), breast cancer, EMT, metastasis, invasion

## Abstract

Metastatic capacities are fundamental features of tumor malignancy. ADP-ribosylation factor (ARF) 1 has emerged as a key regulator of invasion in breast cancer cells. However, the importance of this GTPase, *in vivo*, remains to be demonstrated. We report that ARF1 is highly expressed in breast tumors of the most aggressive and advanced subtypes. Furthermore, we show that lowered expression of ARF1 impairs growth of primary tumors and inhibits lung metastasis in a murine xenograft model. To understand how ARF1 contributes to invasiveness, we used a poorly invasive breast cancer cell line, MCF7 (ER^+^), and examined the effects of overexpressing ARF1 to levels similar to that found in invasive cell lines. We demonstrate that ARF1 overexpression leads to the epithelial-mesenchymal transition (EMT). Mechanistically, ARF1 controls cell–cell adhesion through ß-catenin and E-cadherin, oncogenic Ras activation and expression of EMT inducers. We further show that ARF1 overexpression enhances invasion, proliferation and resistance to a chemotherapeutic agent. *In vivo*, ARF1 overexpressing MCF7 cells are able to form more metastases to the lung. Overall, our findings demonstrate that ARF1 is a molecular switch for cancer progression and thus suggest that limiting the expression/activation of this GTPase could help improve outcome for breast cancer patients.

## INTRODUCTION

To this day, there is no effective targeted therapy for triple-negative breast cancers (TNBC). This breast cancer subtype typically possesses several characteristic features including a basal-like phenotype, lack of expression of ER, PR and HER2 and a poor clinical outcome due to visceral metastasis. TNBC are highly heterogeneous, numerous key genes and proteins have been identified as potential molecular targets. For example, expression of the Epidermal Growth Factor Receptor (EGFR) is increased in more than 50% of TNBC and is associated with proliferation and invasion engaging mitogenic and survival pathways [1-3]. Although EGFR inhibitors have been developed, they have shown limited effects when used alone, mainly due to the development of resistance. Because TNBC show an aggressive pattern of progression with a high rate of early-occurring metastasis, the need for an effective and targeted therapy is therefore urgent.

The important role of small GTP-binding proteins, in the progression of cancer, has been first demonstrated by the discovery of the Ras oncogene and the signaling pathways it engages [4-6]. We and others have shown that the Ras-related ADP-ribosylation factors (ARF) are another class of small GTPases regulating key features of cancer cells [7-11]. Two of the six identified isoforms, ARF1 and ARF6, are the best characterized. ARF6 has been shown to localize to the plasma membrane, regulating receptor endocytosis, membrane lipid transformation and remodeling of the actin cytoskeleton [7, 12]. In contrast, ARF1 has been classically associated with the Golgi apparatus and first identified as a key molecular switch responsible for vesicular transport through early compartments of the secretory pathway [13]. However, in some cell types, this isoform can also be found at the plasma membrane [8, 14-16]. Activation of ARF proteins requires guanine nucleotide exchange factors (ARF GEF), while inactivation is facilitated by GTPase-activating proteins (ARF GAP). The expression of both ARF1 and ARF6 is up-regulated in the most invasive breast cancer cell lines [8, 12]. Using mutants mimicking the active and inactive forms, as well as RNA interference to knockdown expression of these ARF isoform, ARF6 was first identified as a key regulator of invasiveness [7]. Furthermore, silencing of the ARF GEF, GEP100, was found to be an effective strategy to inhibit lung metastasis in mice suggesting that *in vivo*, ARF GTPases may contribute to the progression of breast cancer [17].

We have previously shown that ARF1 activation, following EGFR stimulation, plays a key role in TNBC cell invasion [10]. However, whether ARF1 is important for tumor progression, *in vivo*, has never been examined. Our findings reveal the importance of ARF1 in the most lethal forms of breast cancer and identify this GTPase as a potential new target for the design of next generation breast cancer treatments.

## RESULTS

### Enhanced ARF1 expression in breast cancer tissue samples correlates with poor patient prognostic

**Figure 1:**
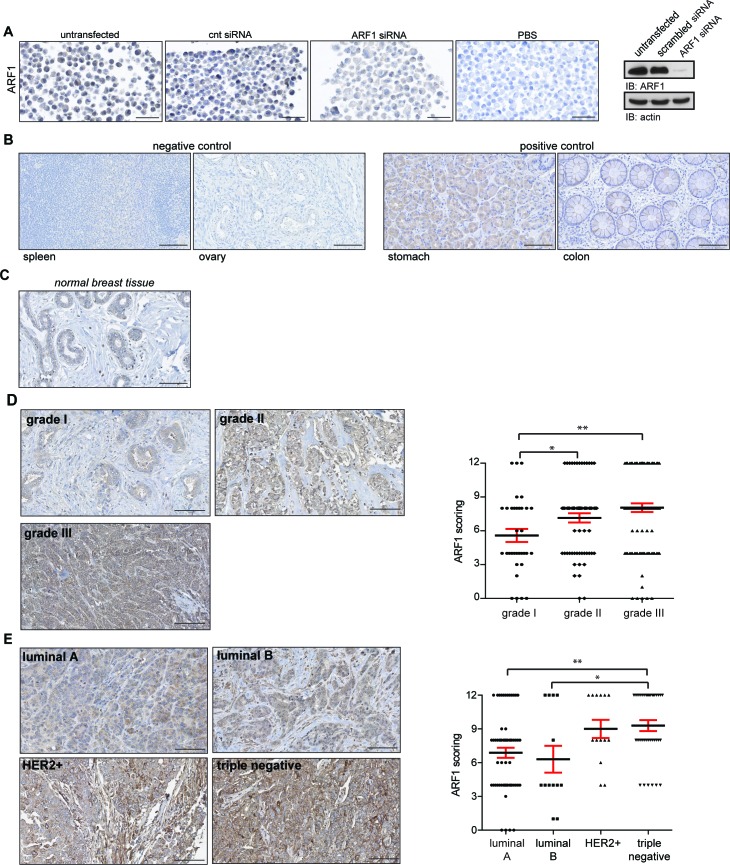
ARF1 expression correlates with molecular subtypes of breast cancer and is associated with tumor grade **A.** MDA-MB-231 cells were transfected or not with (cnt; scrambled) or ARF1 siRNA. Each pellet of cells was incubated in the presence or the absence of the anti-ARF1 antibody. Scale bar, 50 μm. Endogenous level of ARF1 and actin were analyzed by Western blot (right panel). **B.** Representative IHC labeling with ARF1 in normal human tissue. Spleen and ovary were chosen as negative controls, while stomach and colon served as positive controls. Each sample was incubated with anti-ARF1 antibody as shown in A. Scale bar, 100 μm. **C.** Expression of ARF1 in normal breast tissues. Samples correspond to individual breast tissue from the same TMA and incubated with anti-ARF1 antibody. Scale bars, 100 μm. **D.** ARF1 expression in breast cancers tissue samples according to histological grades. Samples are from the same TMA described as in A. Scale bars, 100 μm. Graph showing ARF1 labeling intensity in breast cancer tissue samples according to histological grade. Grade I *n* = 34, II *n* = 64 and III *n* = 100, dataset including 198 patients. **E.** Expression of ARF1 in different molecular subtypes of breast cancer. Samples correspond to individual breast cancer tissue from the same TMA and incubated with anti-ARF1 antibody. Scale bars, 100 μm. Graph depicting ARF1 labeling intensity of breast cancer tissue samples according to molecular subtype. Luminal A *n* = 60, luminal B *n* = 13, HER2+ n *n* = 14 and triple-negative *n* = 37, dataset including 124 patients. In D and E, significance was measured by one-way ANOVA followed by Tukey's multiple comparison tests. * *p* < 0.05, ** *p* < 0.01.

To study the relevance of ARF1 in breast cancer, we aimed to examine whether expression of this GTPase was modulated in tissue samples from breast cancer patients. First, we validated the specificity of the IHC reaction with the ARF1 antibody. MDA-MB-231 cells were used because we have previously shown that they strongly labeled with the ARF1 antibody. As illustrated in Figure 1A, cells were left intact, or transfected with either a scrambled or ARF1 siRNA. Strong labeling was observed in intact and siRNA scrambled conditions. However, ARF1 depletion led to an almost complete loss of immune reactivity. An adjacent section from the same untransfected cells was incubated with a non-immune serum that contained IgG (same isotype/same species) showing complete lack of expression of ARF1. These data confirmed the specificity and sensitivity of our approach before TMA experiments were undertaken.

To examine levels of ARF1 expression in human cancer, we first used negative (spleen and ovary) and positive (stomach and colon) controls as predicted by the literature, and in accordance with the Human Protein Atlas [http://www.proteinatlas.org] [18]. As illustrated in Figure 1B, immunostaining conditions were satisfactorily established. Then, we controlled the labeling of ARF1 in normal breast tissue (Figure 1C). We next investigated ARF1 expression in human breast cancer tissue of various histological grades. We found a positive correlation between elevated levels of ARF1 and breast cancer of higher histological grades (Figure 1D). Finally, we examined the presence of ARF1 in a TMA comprising a variety of breast cancer tissues. Variations in the levels of ARF1 expression according to molecular subtypes of breast cancer were assessed. Although all subtypes were found to be positive for ARF1, samples collected from patients with luminal A and luminal B breast cancer had the lowest level of this ARF isoform. In sharp contrast, both HER2-positive and TNBC subtypes demonstrated higher levels of ARF1, even though only TNBC showed to be significantly different from luminal breast tissue (Figure 1E). We also examined levels of ARF6 proteins. First, expression of this ARF isoform was assessed in negative (heart muscle and skin dermis) and positive (colon and pancreas) controls in accordance with the Human Protein Atlas [http://www.proteinatlas.org] (Suppl Figure 1A). Next, we controlled the labeling of ARF6 in normal breast tissue (Suppl Figure 1B). As expected, we found a positive correlation between elevated levels of ARF6 and breast cancer of higher histological grades (Suppl Figure 1C). Surprisingly, we found that patients with HER2-positive breast cancer had the lowest level of this ARF isoform (Suppl Figure 1D). Luminal A, luminal B and TNBC subtypes demonstrated higher levels of ARF6. Interestingly, level of this ARF isoform was less pronounced than ARF1 in TNBC (Figure 1E and Suppl Figure 1D).

Altogether, our results indicate that overexpression of ARF1 is closely associated with the most lethal and advanced forms of breast cancers.

### ARF1 expression controls the formation of primary tumors and metastases *in vivo*

To investigate the role of ARF1 in tumor formation and metastasis, we generated a human TNBC cell line expressing an inducible control (scrambled) or ARF1 shRNA to trigger the knockdown of this ARF isoform. MDA-MB-231 cells were first infected with lentiviruses containing scrambled or ARF1 shRNA sequences. A 3-day treatment of the cells with doxycycline (dox) blocked the ability of an EGF stimulation to engage signaling *via* the PI3K pathway without affecting the ability of the receptor itself to become phosphorylated or signal through the Erk1/2 pathway (Suppl Figure 2A). These observations correlate with our previous findings where depletion of ARF1 using siRNA only reduced EGFR signaling to the survival pathway [8]. Furthermore, we examined whether our shRNA was effective in suppressing expression of the GTPase over a long period of time. As illustrated in Suppl Figure 2B, induction of the shRNA with dox was effective in inhibiting expression of ARF1 well over a month.

**Figure 2:**
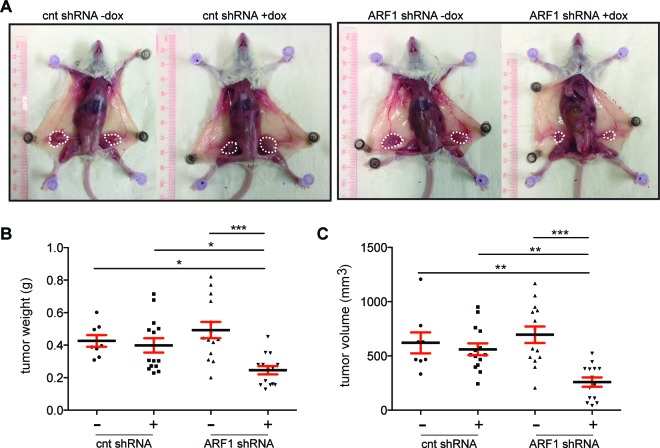
ARF1 regulates primary invasive breast cancer tumor in a mouse model **A.** Primary tumour growth was measured upon orthotopic injection of MDA-MB-231 cells with control (cnt; scrambled) or ARF1 shRNA in SCIB/beige mice, +/− doxycycline (dox). Representative tumors are shown in different panels, *n* = 8, 14, 14, 14 tumors per group, respectively. **B.** Graph showing quantitative tumor weight of each group. **C.** Quantitative results of tumor volume. **B.** and **C.** Significance was measured by one-way ANOVA followed by Tukey's multiple comparison tests. * *p* < 0.05, ** *p* < 0.01, **** *p* < 0.0001.

Cells were injected orthotopically into the fourth mammary fat pads of severe combined immunodeficiency mice (SCID)/beige female mice and tumor growth was monitored weekly (Figure 2). Once development of primary tumor masses became visible, mice were randomly separated to receive food, which contained or not dox. Eight weeks after implantation and in conditions where ARF1 expression was inhibited, primary tumors were smaller than the controls (scrambled shRNA -/+ dox and ARF1 shRNA - dox) (Figure 2A). Knockdown of ARF1 affected tumor growth by reducing tumor weight and volume (Figure 2B, 2C). Lungs and brain were also analyzed by gross examination and no metastatic lesions were found.

**Figure 3:**
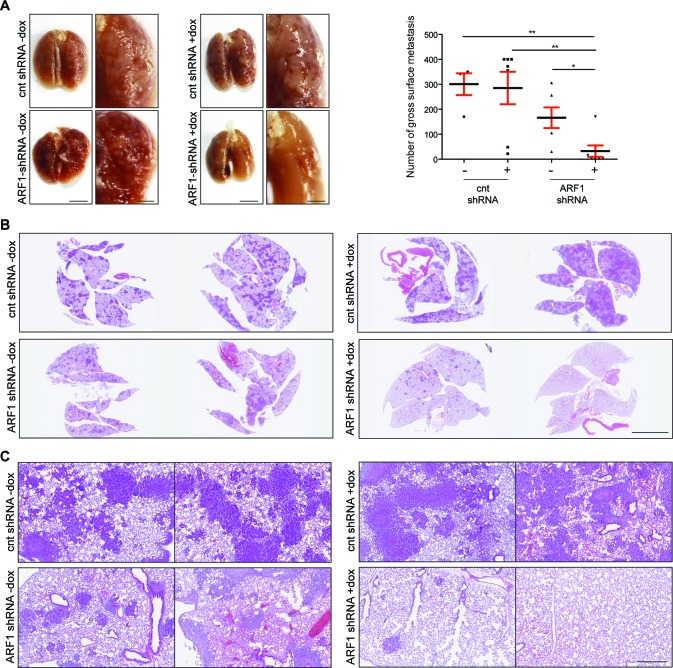
ARF1 depletion impaired breast mouse metastasis **A.** Gross images of SCID/beige lungs, two months after tail vein injection of MDA-MB-231 cells with control (cnt; scrambled) or ARF1 knockdown, +/− doxycycline (dox). *n* = 4, 7, 6 and 7 mice per group respectively. Scale bars, 1 cm. Graph on the right shows quantitative results of gross surface lung metastases. Significance was measured by one-way ANOVA followed by Tukey's multiple comparison tests. * *p* < 0.05, ** *p* < 0.01. **B.** Histological section of lungs from SCID/beige mice bearing metastatic foci. Scale bars, 5 mm. **C.** Higher magnification from **B.** of H&E stained sections of the lungs. Scale bars, 500 μm.

The effect of ARF1 depletion on the metastatic potential was next investigated by injecting the cells into the mouse lateral tail vein. Metastatic colonization was evaluated after two months by gross examination and microscopic inspection of tissue sections. As illustrated in Figure 3A, multiple metastatic lung lesions were observed in mice injected with control MDA-MB-231 cells. However, when expression of ARF1 was suppressed, only a few metastases where noticed at the lung surface. The lungs from each group were then removed and processed for histological examination. The number of metastatic clusters, present in the lungs of mice injected with scrambled shRNA cells was significantly higher than that in the ARF1 group injected with ARF1-shRNA cells (Figure 3B). Microscopic examination of lung tissue sections revealed a sharp decrease in the number of metastasic foci of the lung when ARF1 expression was knocked down (Figure 3C). In each group, all metastases were found outside of the vessels. A visual inspection of other organs was made and showed no evidence of metastasis.

Taken together, these data demonstrate that inhibition of ARF1 expression, in highly invasive breast cancer cells, decreased both primary breast tumor formation and metastatic breast tumors within the lung.

### ARF1 overexpression promotes EMT of human breast cancer cells

To address whether overexpression of ARF1 is sufficient to confer enhanced invasive capacities of breast cancer cells, we next used the ER^+^ MCF7 cells characterized as highly proliferative, but non-invasive tumor cells. These cells have low metastatic potential *in vivo* [19] and express low levels of both ARF1 and ARF6 when compared to MDA-MB-231 cells (Suppl Figure 3A). First, we confirmed that both ARF isoforms could be activated in MCF7 cells. As expected, FBS treatment enhanced ARF1 activation by a 2.1-fold and ARF6 by 3-fold compared to control conditions (Suppl Figure 3B). It was previously suggested that, in Hela cells, activation of ARF1 was under the control of ARF6 [15]. To determine whether, in our model, ARF6 had any influence on ARF1 activity, we first overexpressed the different ARF isoforms and looked at their activation (Suppl Figure 3C). We found that the activation of each ARF, in MCF7 cells, is not affected by the overexpression of the other isoform. We confirmed these data by depleting ARF6 and assessing ARF1 activity. Under unstimulated or stimulated FBS conditions, ARF1 activation was not affected by ARF6 knock down (Suppl Figure 3D).

**Figure 4:**
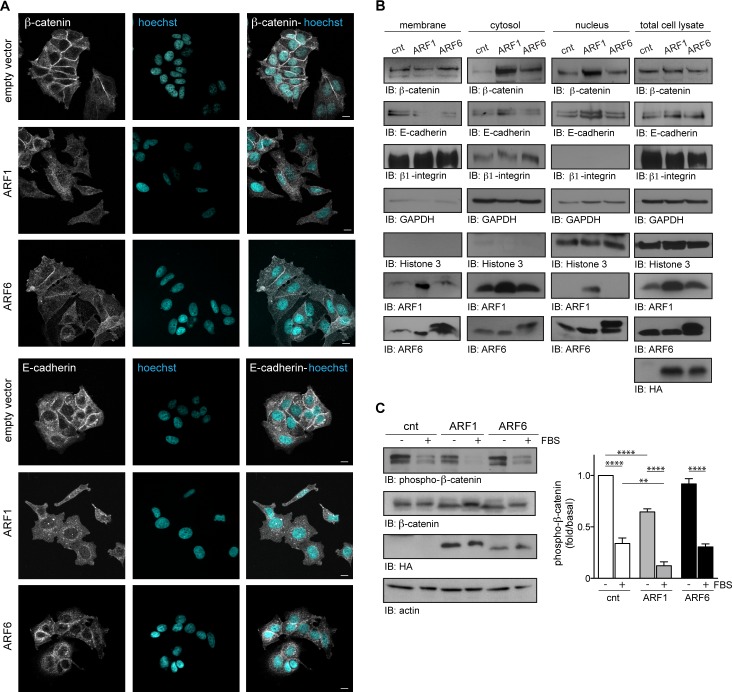
ARF1 is important for the maintenance of adherent junctions **A.** MCF7 cells were transfected with empty vector (cnt), ARF1 or ARF6 and fixed after 48 hours. Cells were stained for β-catenin, E-cadherin and nuclei with Hoechst. Images are from three independent experiments, with more than 100 cells per condition. Scale bars, 10 μm. **B.** Cells transfected as in **A.** were used to prepare membrane fractions. Associated β-catenin, E-cadherin, ARF1 and ARF6 were assessed by Western blotting. These experiments are representative of three others. β1-Integrin was used as a plasma membrane marker, GAPDH as a cytosol marker and Histone 3 as nuclei marker. **C.** Cells transfected as in **A.** were stimulated with FBS (10%) for four hours. Endogenously expressed β-catenin, HA-tagged (hemagglutinin) proteins and actin were detected by Western blotting. Data are the mean ± SEM of four experiments. Statistical analysis was performed using a two-ways ANOVA followed by a Bonferroni's multiple comparison test. **p < 0.01, ****p < 0.0001.

MCF7 cells characteristically proliferate maintaining tight cell-cell junctions. As illustrated in Figure 4, β-catenin and E-cadherin localization to the cell membrane serves to maintain this non-invasive phenotype observed in various carcinomas. In conditions where ARF1 was overexpressed, MCF7 cells exhibited a spindle-like mesenchymal morphology and membrane localization of β-catenin and E-cadherin was lost (Figure 4A). ARF6-overexpressing cells maintained their epithelial morphology, but less β-catenin and E-cadherin were present at the cell-cell junctions (Figure 4A). Plasma membrane, cytosolic and nuclear fractions were isolated biochemically to compare the amount of ARF1 in each cellular fraction. Overexpression of the GTPases caused their relocalization to different cellular compartments, but increased strongly their localization to the plasma membrane supporting additional roles, when overexpressed (Figure 4B). Western blot analysis revealed that total expression of β-catenin and E-cadherin was not affected by ARF overexpression (Figure 4B). However, both β-catenin and E-cadherin levels were diminished in ARF1-overexpressing MCF7 cellular membrane fractions, but found enhanced in cytosolic and nuclear fractions. Similarly, overexpression of ARF6 also resulted in increased cytosolic β-catenin, and diminished E-cadherin in membrane fractions (Figure 4B). Because localization of β-catenin is associated with its activity, we next examined its phosphorylation state. Expression of ARF1 spontaneously decreased the basal level of β-catenin phosphorylation (Ser^33^, Ser^37^, Thr^41^) by 1.7 fold compared to control cells. However, when cells were in the presence of FBS, phosphorylation of ß-catenin was markedly decreased reflecting an activation of this signaling protein (Figure 4C). In contrast, ARF6 expression did not have a significant effect. These results therefore provide evidences that ARF1 is a crucial regulator of cell-cell adhesion by repressing adherent junctions formation through a ß-catenin-dependent pathway and epithelial tight junctions through E-cadherin.

**Figure 5:**
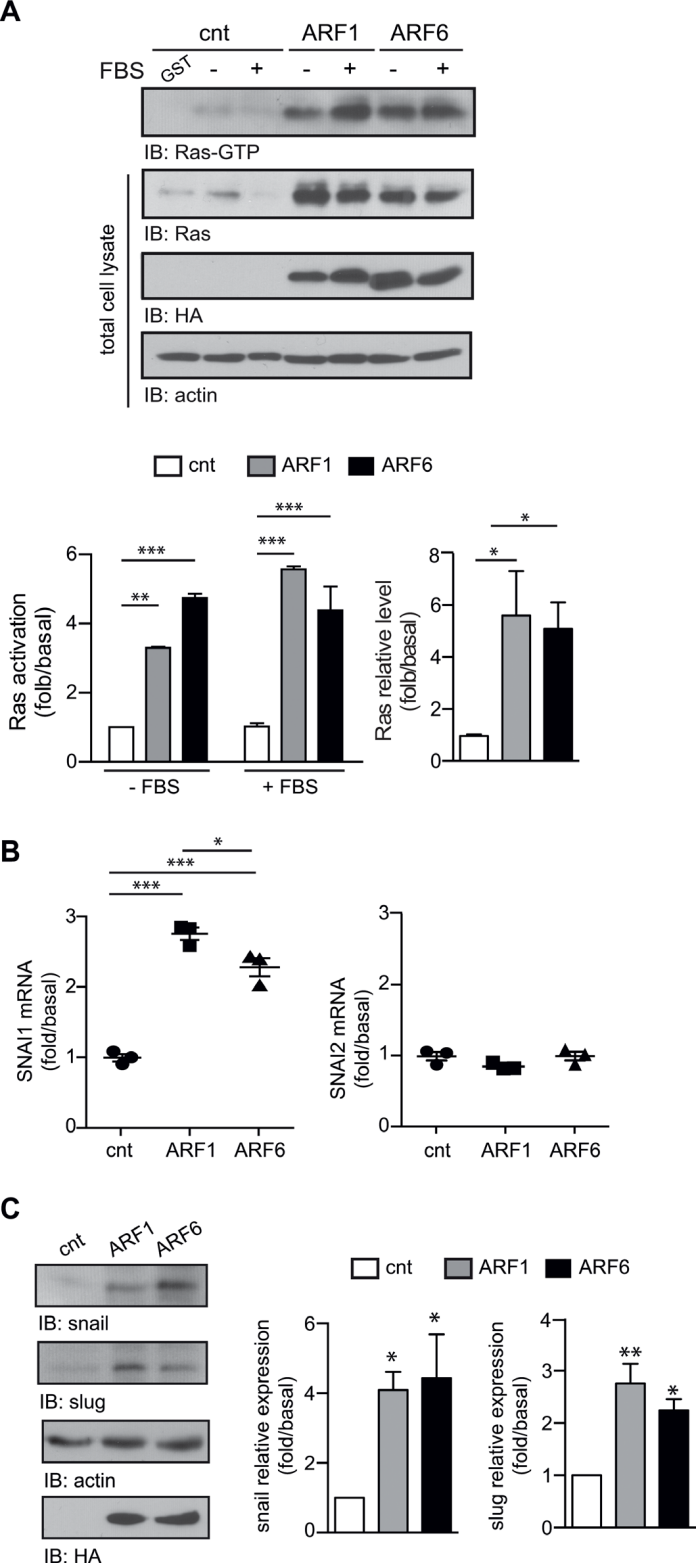
ARF proteins control key factors of EMT **A.**,**B.**, **C.** Cells transfected with empty vector (cnt), ARF1 or ARF6. **A.** Cells were stimulated with FBS (10%) for four hours. Ras-GTP levels were assessed by Western blot analysis. Levels of Ras, actin and HA tagged proteins were determined by Western blot. Left graph is representative of Ras activation. Data are the mean ± SEM of four experiments. Statistical analysis was performed using a two-ways ANOVA followed by a Bonferroni's multiple comparison test. **p < 0.01, ***p < 0.001. Right graph is representative of Ras expression level. Data are the mean ± SEM of five experiments. Statistical analysis was performed using a one-way ANOVA followed by a Dunnett's multiple comparison test. *p < 0.05. **B.** Total RNA was extracted from MCF7 and SNAI1, SNAI2 mRNA were analyzed. Experiments are representative of three experiments performed in triplicate. *p < 0.05, ***p < 0.001. **C.** Levels of snail and slug protein were determined by Western blot. Data are the mean ± SEM of three experiments. Statistical analyses were performed using a one-way ANOVA followed by a Bonferroni's multiple comparison tests. *p < 0.05, **p < 0.01.

Alternatively, activation of Ras can induce EMT by enhancing the expression of a panel of transcription factors such as snail (*SNAI1*) and slug (*SNAI2*) [20]. We next determined whether the overexpression of ARF1 could directly impact Ras signaling since MCF7 cells do not exhibit activating mutation of this well-characterized oncogene. As depicted in Figure 5A, overexpression of ARF1 enhanced basal expression and activation of this prototypical GTPase. Similar findings were obtained following expression of ARF6. Furthermore, overexpression of either ARF proteins enhanced mRNA and protein levels of SNAI1/snail (Figure 5B and 5C). In contrast, mRNA levels of SNAI2 was unaffected by overexpression of ARF1 or ARF6, but higher protein levels of slug was found in both conditions. These results therefore suggest that high expression of ARF proteins can contribute to EMT by controlling activation of classic inducers such as oncogenic Ras, snail and slug.

### ARF1 overexpression promotes cellular motility, invasiveness, proliferation and survival

**Figure 6:**
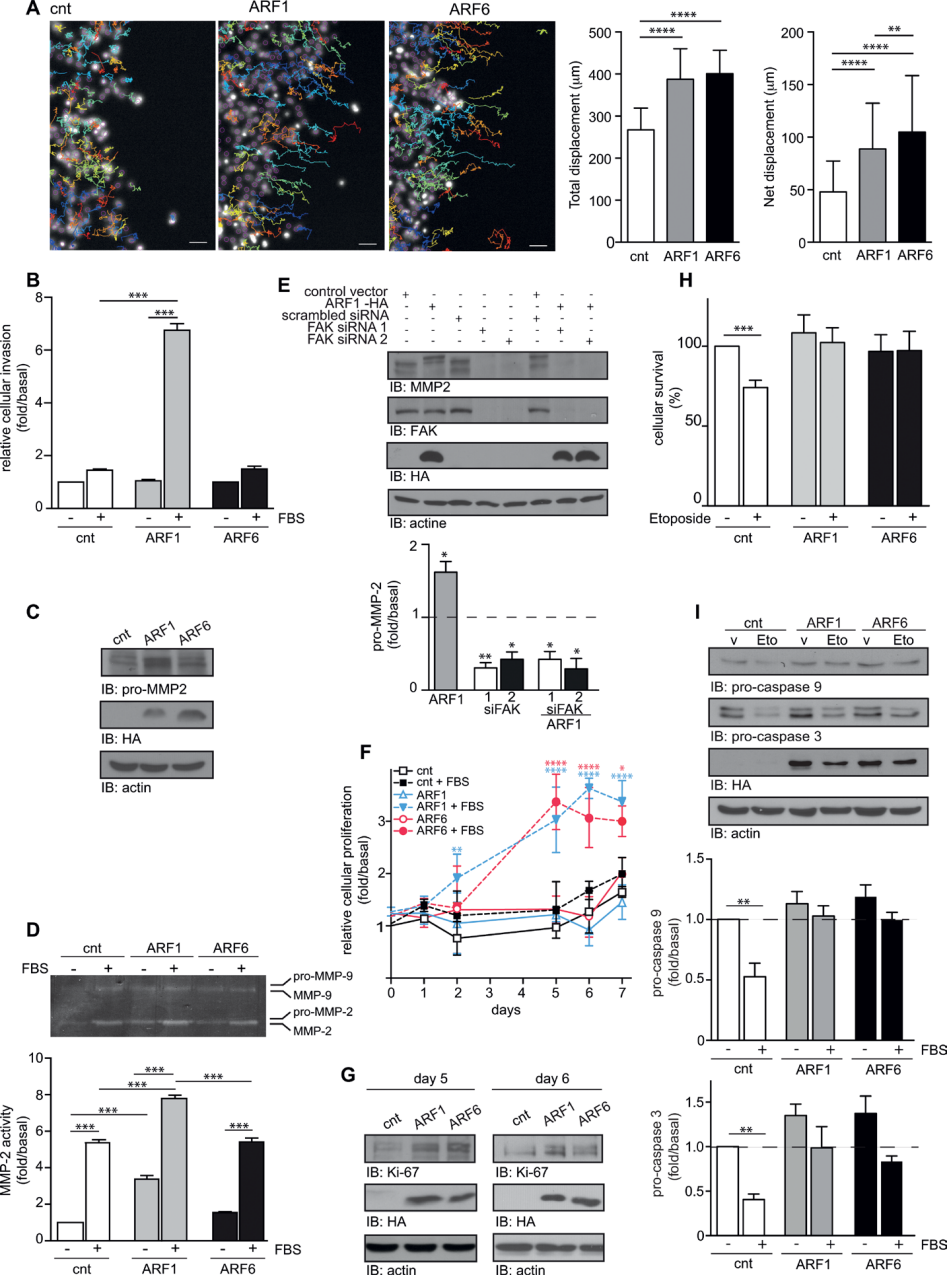
Overexpression of ARF controls motility, invasion, proliferation, and survival of MCF7 cells **A.** MCF7 were transfected with empty vector (cnt), or ARF1 or ARF6. After 48h, confluent cells were scratch and wound healing was monitored over 16h in the presence FBS. Hoechst-stained nuclei were tracked using Fiji software. Representative pictures of cells tracking over 16h are shown. Scale bars, 50 μm. Quantification of cell motility: right graphs represent the mean total and net displacement. *n* = 120-150 cells. **p < 0.001,****p < 0.0005. **B.** Cells transfected as in A, were seeded into Matrigel-coated Boyden chambers. Cells were left untreated or stimulated with FBS 10% for 16 hours. Graph is representative of three images taken, per condition, of three independent experiments. **C.** Protein expression of pro-MMP2, HA-tagged protein and actin were detected by Western immunoblotting. **D.** Transfected cells were stimulated or not for 12 hours with 10% FBS. Supernatants were collected and analyzed by zymography. Data are the mean ± SEM of three experiments. **E.** Cells were transfected with either empty vector, HA-ARF1, scrambled or two different FAK siRNA. Protein expression of pro-MMP2, FAK, HA-tagged protein and actin were detected by Western immunoblotting. Graph represents the mean ± SEM of five experiments. Significance was measured by one-way ANOVA followed by Bonferroni's multiple comparison tests. *p < 0.05, **p < 0.01. **F.** MCF7 cells transfected as in A, were seeded in 96-well plates. Proliferation was measured by MTT (Thiazolyl blue-tetrazolium-bromide) over 7 days. These experiments are representative of four performed in triplicate. **G.** Endogenous expression of Ki-67, ARF6 and actin was detected by Western immunoblotting, after 5 and 4 days. **H.** Cells were transfected as in **A.** and 24 hours after the transfection, they were treated with vehicle or etoposide. After 48 hours, surviving cells were analyzed by FACS, using Annexin-V binding and PI permeability assays. Data are the mean ± SEM of three experiments respectively. **I.** Transfected cells treated as in **H.** and level of pro-caspase 9, pro-caspase 3, actin and HA-tagged protein were detected by Western immunoblotting. Data are the mean ± SEM of three experiments. In **D.**, **H.** and **I.**, significance was assessed by two-way ANOVA followed by Bonferroni's multiple comparison test. *p < 0.05, **p < 0.01, ***p < 0.001.

We next studied cellular responses associated with EMT. Using time-lapse microscopy, we quantitatively assessed motility of control and ARF1-overexpressing cells using a wounding assay. After 16h in high serum media (10% FBS), when ARF1 was expressed, total and net displacements were enhanced compared to control conditions (Figure 6A). Similar results were found for ARF6-overexpressing cells. However, net displacement was found more enhanced than ARF1 (Figure 6A). To complement these findings, we examined cell invasion using Matrigel-coated Boyden chambers. As illustrated in Figure 6B, FBS stimulation did not significantly increase the ability of MCF7 cells to migrate in this transwell assay. However, when ARF1 was overexpressed and cells stimulated by FBS, the invasive capacity was enhanced by 6-fold. In contrast, overexpression of ARF6 did not have a significant effect in this assay. In these conditions, zymography assay revealed that overexpression of ARF1 could activate a specific metalloproteinase, MMP-2, by controlling its expression *via* focal adhesion kinase (FAK) (Figure 6C, 6D and 6E). These findings further confirm that expression of ARF1 can enhance the invasive capacity of MCF7 cells.

Since we noticed that cellular proliferation was enhanced in ARF1-overexpressing cells, we performed MTT assays to examine proliferation over a 7-day period. Basal proliferation of control, ARF1 and ARF6-overexpressing MCF7 cells was similar. FBS treatment had no marked effect in control condition. Interestingly, this effect was greatly enhanced by FBS treatment when cells overexpressed ARF1 or ARF6 proteins (Figure 6F). In this context, expression of Ki-67, a proliferative marker, was also increased, contributing to the overall effect observed (Figure 6G). We confirmed our results by qualitatively assessing proliferation of control, ARF1 and ARF6-overexpressing cells using a 5 days wounding assay. When control MCF7 cells were left in low serum media (1% FBS), there was no significant closure of the wound, and FBS treatment had no marked effect. When ARF1 was overexpressed, FBS stimulation had a more potent effect and wound healing was observed (Suppl Figure 4). Similar results were found for ARF6-overexpressing cells (Suppl Figure 4).

In order to understand how ARF proteins control these processes, we next looked at signaling pathways regulating motility, invasion and proliferation of cancer cells. Suppl Figure 5 shows that overexpression of ARF1 potentiated activation of the MAPK and PI3K pathways assessed respectively by the phosphorylation of Erk 1/2 and Akt. In contrast, ARF6 overexpression only resulted in the activation of the MAPK pathway.

It has been previously reported that cells with increased invasive phenotypes exhibit decreased sensitivity to apoptotic stimuli [21]. We therefore examined whether ARF1 overexpression may protect against a treatment with the cell death inducer, etoposide. Analysis of apoptosis using the Annexin V/PI assay revealed that treatment of control cells with this topoisomerase inhibitor decreased by 25% cell survival (Figure 6H). Overexpression of ARF1 protected MCF7 cells from etoposide-induced death. Similar results were obtained when ARF6 was overexpressed (Figure 6H). To induce its effect, etoposide is known to promote activation of caspase proteases 9 and 3. As illustrated in Figure 6I, induction of caspase-9 and caspase-3 protease activity, in cells exposed to etoposide, was abolished when cells overexpressed ARF1 or ARF6.

Altogether, these results suggest that overexpression of ARF proteins not only enhances the invasive capacity of MCF7 cells, but also their ability to proliferate and survive.

### Overexpression of ARF is effective to promote metastasis *in vivo*

**Figure 7:**
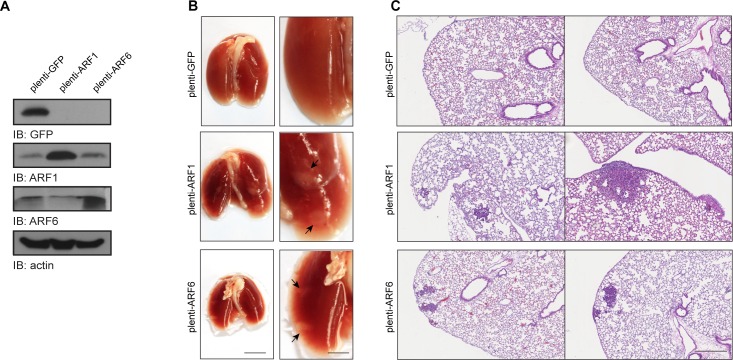
ARF1 and ARF6 overexpression increases metastasis *in vivo* **A.** MCF7 cells stably expressing GFP, ARF1 or ARF6 used for tail vein injection, were lysed and protein expression of GFP, ARF1, ARF6 and actin was detected by Western immunoblotting. **B.** Images of SCID/beige lungs after tail vein injections of MCF7 cells stably expressing GFP, ARF1 or ARF6. *n* = 10, 9 and 10 mice per group, respectively. Arrows indicate metastatic tumor. Scale bars, 1 cm. **C.** H&E stained sections of lungs from mice bearing the indicated tumors; representing metastatic foci. Scale bars, 500 μm.

Because our results suggest that high expression of ARF1 is associated with cancer cell invasiveness, we finally examined whether overexpression of this small GTP-binding protein could confer the ability of non-invasive cancer cells to form metastasis *in vivo*. Control or ARF-overexpressing MCF-7 cells were injected into the tail vein of SCID/beige mice (Figure 7A). Lung metastases were examined after six weeks. Injection of control cancer cells did not form external metastatic nodules to the lungs (Figure 7B). However, when ARF1 was overexpressed, the appearance of tumors was noticed. Microscopic examination of lung tissue sections, by H&E staining, revealed that overexpression of ARF1 led to the formation of internal extravasated metastatic nodules in contrast to the control condition (Figure 7C). An increase of metastatic potential was also found when ARF6 was overexpressed. These results suggest that overexpression of ARF proteins can promote the colonization process of breast cancer cells into the lung and therefore contribute to breast cancer metastasis formation in mice.

## DISCUSSION

In this report, we describe the molecular mechanism by which ARF1 confers an invasive phenotype to breast cancer cells and show that *in vivo*, this key molecular switch greatly contributes to tumorigenesis. We have previously reported that ARF1 plays a major role in controlling the proliferation, migration and invasion of TNBC cells [8, 10]. However, whether in an *in vivo* setting, elevated ARF1 expression, in certain types of breast cancer cells, indeed contributes to tumor invasiveness was unknown. Tumor progression, in animal models and human, is greatly influenced by the stroma through various types of heterotypic signaling capable of altering the aggressive behaviors of carcinoma cells [22]. Therefore, the way tumor cells behave in 2D or 3D-tissue culture doesn't necessarily reflect the way they behave *in vivo*. It is thus important to define the role of the proteins we identify in cellular models as key regulators of cancer cell behaviors in their natural environment in order to validate their key roles and identify them as potential therapeutic targets or new biological markers [23, 24]. In the present study, we provide strong evidences that ARF1 is a key protein of breast cancer invasiveness in a murine model and further demonstrate that it could be a potential prognostic factor for patients.

We have chosen to perform this study with MDA-MB-231 and MFC7 cells, which express high and moderate levels of ARF1, respectively. Using these two well-characterized cell lines, we have examined molecular alterations and *in vivo* tumor progression. Using orthotopic injection of ARF1 knockdown tumor cells into the mammary fat pads of SCID/beige mice, we show that ARF1 is required for primary tumor growth. We also show, using injection in mouse-tail veins, that ARF1 knockdown affects the later steps of the metastasis cascade. Although the stroma contributes to tumor progression, depletion of ARF1, in invasive breast cancer cells, is associated with a significant decrease of malignant potential. Conversely, non-invasive MCF7 cells formed metastases to the lung when levels of ARF1 were enhanced. The demonstration that ARF1 regulates tumorigenesis complements previous reports that highlighted the implication of another ARF isoform in this process. By modulating the activity of ARF6 through knockdown of a nucleotide exchange factor, GEP100, tumor metastasis to the lung was impaired [17]. Furthermore, inhibition of the ARF6 GAP AMAP1 was also reported to limit metastatic activities *in vivo* [25]. Using similar experimental models, our findings directly demonstrate the key role of ARF1 in metastasis. To support this, we show here that in tissue samples from breast cancer patients, overall expression level of ARF1 is correlated with the most aggressive subtypes of tumor and high-grade breast cancer. We have found similar results for ARF6 when examining tumor grade. However, overexpression of this isoform differed from ARF1 according to the molecular subtypes. Our IHC data for ARF6 are supported by previous studies performed on a breast cancer TMA that showed an increase of ARF6 level in breast tumor metastasis cores and up-regulation, at the plasma membrane, in high-grade triple-negative breast cancers [26, 27].

To better understand how ARF1 may contribute to invasiveness, we overexpressed the GTPase into a cell model known to be poorly invasive. MCF7 cells have conserved several characteristics of differentiated mammary epithelium, namely the ability to form tight cellular junction similar to those occurring in normal epithelial cells and therefore to grow as interconnected colonies in 2D tissue culture. Our findings demonstrate that overexpressing ARF1 leads to major phenotypical changes. First, cells lose their epithelial characteristics and acquire a mesenchymal phenotype with the hallmarks of EMT. We show that key inducers such as the transcription factors snail and slug are also upregulated. Furthermore, membrane E-cadherin is lost and ß-catenin is activated. This process was previously shown to impact E-cadherin localization. When phosphorylated on specific residues, ß-catenin is largely defective in E-cadherin binding [28-31]. It was previously reported that changes in ß-catenin localization are not always associated with changes in its expression, further supporting the importance of examining ß-catenin localization and phosphorylation state [32]. ARF1 overexpression also led to activation of several signaling events also known to contribute to EMT (Ras, Erk 1/2, PI3K/Akt pathway). Together, our findings demonstrate that ARF1 controls ß-catenin and E-cadherin function, possibly by inhibiting β-catenin phosphorylation through its ability to potentiate Ras signaling. Although ARF6 can promote Ras activation, this isoform only modulate ß-catenin localization. Furthermore, when ARF1 is overexpressed in MCF7, it has the potential to activate, not only the MAPK pathway, like ARF6, but both the MAPK and PI3K pathways. These results support our previous work in invasive cell models where ARF1 acts to control Rho signaling, well known for its role in EMT [33, 34]. Furthermore, we recently reported that ARF1 controls activation of metalloproteinases, key proteins of EMT [10, 35]. We also demonstrated that ARF1 controls FAK activity, a key protein for MMP expression [11, 36-38]. Here, we show that overexpression of ARF1 in MCF7 could activate MMP-2 by controlling its expression *via* FAK, a process which may contribute to the newly acquired invasive phenotype. We also demonstrate that ARF1 and ARF6 control major processes that increase tumor metastasis *in vivo* such as cell motility, migration, proliferation, and resistance to apoptosis.

In summary, we provide evidence that ARF1 is a key regulator of cancer progression. For the first time, we demonstrate that elevated level of ARF1 is associated with a higher incidence of metastases and that its expression is elevated in high-grade cancers and triple-negative basal-like breast cancers. These results support the use of ARF1 as a potential pharmacological target for invasive breast cancer therapy.

## MATERIALS AND METHODS

### Patients, tissue samples and Tissue microarray (TMA)

A retrospective study was carried out using a cohort of 198 female breast cancer patients comprising tumors of different histological grades [39]. Archived formalin-fixed paraffin-embedded (FFPE) samples containing tumor tissues were collected for the study. Tumor grades from each breast cancer (FFPE) samples were confirmed using the Modified Scarff-Bloom-Richardson-Elston-Ellis grading system (SBR-EE) [40]. In addition, extraneous tissues such as spleen, ovary, stomach and colon were included in this study as control. All samples were obtained from Centre Hospitalier de l'Université de Montréal (CHUM) after granting the approval of the research ethical committee (Approval No. SL 05.019). Tissue microarrays were next constructed as previously described [39]. Sections (4 μm) from each paraffin block were stained with hematoxylin and eosin (H&E) and examined by two independent pathologists. Core punches, 1 mm in diameter, were drilled from representative areas contained within each FFPE tumor blocks. Each core was realigned in duplicate or triplicate into recipient blocks according to the intended design of the map using a Manual Tissue Arrayer I (Beecher Instruments). Blocks were next inverted and incubated overnight in the oven over a glass slide. TMA blocks were allowed to cool until they could easily detach from the glass slide. Tissue sections from each TMA were prepared and one slide from each block was stained with H&E to review the diagnosis and histological grades on all tissue samples. Additional representative sections from each block were submitted to automated immunohistochemical (IHC) staining.

### Immunohistochemistry (IHC)

IHC was performed on TMA containing representative FFPE tumor tissue samples. Anti-ARF1 antibody (dilution 1/350) was applied to every section for 2 hours at room temperature. Sections were then incubated with a specific secondary biotinylated antibody. Streptavidin horseradish peroxidase, and 3,3-diaminobenzidine were used according to the manufacturer's instructions (DABmap detection kit, Ventana Medical Systems). Sections were next counterstained with Gill's hematoxylin and sodium bicarbonate. Finally, each slide was coverslipped and scanned at high resolution (40X) using the Nanozoomer Digital Pathology equipment (Hamamatsu, Bridgewater, NJ). Two independent pathologists reviewed all stained sections on two separate occasions.

To assure that labeling conditions with the anti-ARF1 rabbit polyclonal antibody were optimal, assessment of ARF1 expression was made on cell lines first. These assays were carried out according to the manufacturer recommendations on an automated immunostainer (Discovery XT system, Ventana Medical Systems, Tucson, AZ, USA). Heat-induced epitope retrieval was performed with proprietary reagents. Blockers were applied for 8 minutes before the primary antibody to block the endogenous peroxidase activity. Anti-ARF1 antibody (Proteintech) was applied for 2 hours at room temperature. ARF6 antigen recovery was conducted using heat retrieval (Heat-Induced Epitope Retrieval) with standard CC2 (Ventana Medical Systems) using a low pH citrate buffer. Slides were incubated with 1/50 of anti-ARF6 antibody for 3 hours at room temperature. In addition, immunohistochemical analysis of estrogen receptor (ER; monoclonal, clone SP1, RTU, sCC1, Ventana Medical Systems), progesterone receptor (PR; monoclonal, clone 1E2, RTU, sCC1, Ventana Medical Systems), HER2 (monoclonal, clone 4B5, RTU, sCC1, Ventana Medical Systems), Ki-67 (monoclonal, clone SP6, pretreated sCC1, BioCare medical) were used as surrogate markers of breast cancer molecular subtypes. [41]. Antigen retrieval was performed with proprietary reagents followed by incubation with the primary antibody then secondary biotinylated antibody was applied. Streptavidin horseradish peroxidase, and 3,3-diaminobenzidine were used according to the manufacturer's instructions (DABmap detection kit, Ventana Medical Systems). Sections were next counterstained with Gill's hematoxylin and sodium bicarbonate. Finally, each slide was coverslipped and scanned at high resolution (40X) using the Nanozoomer Digital Pathology equipment (Hamamatsu, Bridgewater, NJ).

Scoring of ARF1 and ARF6 expression on each core was carried out using a two tier scoring system as previously described [39]. The first parameter corresponds to the percentage of immunoreactive cells also known as the quantity score (QS). QS was estimated as follows: no staining was scored as 0, 1-10% of cells with positive staining were scored as 1, > 10- 50% as 2, > 50-70% as 3, and > 70-100% as 4. We next assessed the second parameter (staining intensity score), which was rated as follows: No staining → 0, weak staining → 1, moderate staining → 2, and strong staining → 3. The product of the quantity and the staining intensity scores represents the total IHC score that ranges from 0 to 12 [42, 43]. IHC staining according to the College of American Pathologists (CAP)-approved scoring system for ER, PR, HER2 and Ki-67 were used as surrogate markers to classify breast cancer tumors into luminal A, luminal B, HER-2 positive and triple-negative breast cancer [39, 41].

### Cell culture

MDA-MB-231 cells were obtained from ATCC and maintained in DMEM supplemented with 10% fetal bovine serum, penicillin/streptomycin (Wisent, St-Bruno, Canada). MCF7 were obtained from Sylvie Mader (University of Montreal, Canada). Cells were maintained at 37°C in 5% CO2. Cells were transfected with DNA and/or siRNA using Lipofectamine 2000 according to the manufacturer's instructions. ARF1 siRNA was designed against part of the 3′ untranslated region and coding region of ARF1. siRNA corresponding to human FAK (J-003164-16) siFAK1 or (J-003164-14) siFAK2 were used in ours experiments. ARF1, FAK and scrambled siRNA were synthesized by Thermo Science Dharmacon (Lafayette, CO) and used in previous studies [10, 11].

### Antibodies

Anti-ARF1 and anti-ARF6 were from Proteintech (Chicago, IL). Anti-pan-actin, anti-phospho-AKT, anti-AKT, anti- pro-caspase 9, anti-pro-caspase 3, anti-EGFR, anti-phospho-ERK and anti-pro-MMP2 were all from Cell Signaling Technology (Danvers, MA). Anti-ARF6, Anti-ERK, anti-FAK, anti-snail, anti-slug and anti-p-tyrosine were from Santa Cruz Biotechnology (Santa Cruz, CA). Anti-HA was from Abcam (Cambridge, MA). Anti-TurboGFP was from Thermo Fisher Scientific (Rockford, IL) and anti-Ki-67 was from BD Bioscience (Bedford, MA). HRP-conjugated secondary antibodies were from R & D Systems (Minneapolis, MN).

### Lentiviral constructs and virus production

MISSION ARF1 shRNA plasmids were purchased from Sigma Aldrich (ARF1: TRCN0000039873, TRCN0000039874, TRCN0000039875, TRCN0000039876 and TRCN0000039877, ARF6: TRCN0000381410, TRCN0000286788, TRCN0000380270, TRCN0000294067 and TRCN0000294069 and scrambled (SHC016)). The ARF1 TRCN0000039876 and ARF6 TRCN0000380270 target sequences were chosen as they showed the highest level of knockdown in MDA-MB-231 cells (data not shown). ARF1 shRNA oligo duplex sequence and scrambled duplex were from Integrated DNA Technologies (IDT, Coralville, Iowa) and annealed in the doxycycline-inducible pLKO-TET-ON vector (Addgene plasmid #21915) according to Wiederschain protocols [44] [45]. Positive bacterial clones were selected with carbenicillin and sequenced to confirm the identity of shRNA constructs (Genomic platform at the Institut of Research in Immunology and Cancer, Université de Montréal, Montreal, Canada). Lentivirus containing the shRNA were generated using 293T cells transfected with pLKO-TET-ON-ARF1 or scrambled shRNA constructs and the psPax.2 and pMD2.G packaging plasmids. MDA-MB-231 cells were infected in the presence of polybrene (8 μg/ml) and stable clones were selected in the presence of 0.75 μg/ml of puromycin. pLKO-TET-ON-ARF1-shRNA or scrambled-infected MDA-MB-231 cells were maintained in 0.75 μg/ml of puromycin in a tetracyclin-free media. For the generation of MCF7-overexpressing cells, the ARF1, ARF6 or GFP sequence was cloned into the pLenti vector (Addgene plasmid 17448) and lentivirus were generated as described above. Cells were infected and stable clones were selected using 1 μg/ml puromycin.

### Animal protocols

The local institutional animal ethics committee approved all animal studies. For xenograft studies, 6-wk-old SCID/beige female mice were obtained from Charles River Inc. (St. Constant, Quebec, Canada). Before inoculation, pLKO-TET-ON-scrambled-shRNA or pLKO-TET-ON-ARF1-shRNA MDA-MB-231-infected MDA-MB-231 cells grown in serum containing culture medium were washed with PBS buffer. Cell pellets (2 ×10^6^ cells) were resuspended in 50 μl of 1:1 Matrigel (BD Bioscience) plus PBS and injected into the fourth mammary fat pads (MFP) of both flank of the anesthetized mice, using a 27mm gauge needle [46]. All animals were numbered and kept separately in a temperature-controlled room on a 12 hours light/12 hours dark schedule. After the injection of the cells, mice were monitored twice weekly for the development of primary tumor masses. Once the tumors became visible (2 weeks post-injection), mice were randomly separated so that each experimental group was homogeneous to start. Mice received food, which contained or not doxycycline (2018, 625 Doxycycline, Harlan Laboratories, Madison, WI) for shRNA induction [47]. They were weighted and masses were measured two times a week using a caliper. The tumor volume was estimated using the equation volume = π (length) (width^2^)/6 [48]. Two month after the injection, mice were euthanized with CO_2_. A visual examination of the brain and lung found no metastasis. For the experimental metastasis assay, 3 ×10^6^ stable MCF7 or 2 ×10^6^ scrambled or ARF1 shRNA MDA-MB-231 tumor cells were suspended in 200 μl of PBS and were injected into the tail vein of SCID/beige mice [46, 49]. Cell pellets were suspended in 200 μl of PBS. Mice were weighted twice a week. After 6- to 8-weeks, mice were euthanized and lungs were excised. A visual examination of other organs showed no other metastasis. The lungs were dissected from the mice and stored in formalin (10%) solution prior to the counting of visible tumors on all surfaces of the lungs. For metastatic nodules observation, all dissected lungs were paraffin-embedded, sectioned, stained with H&E, and metastatic nodules were microscopically examined. Each slide was scanned at high resolution (40X) using the Nanozoomer Digital Pathology equipment (Hamamatsu, Bridgewater, NJ, USA).

### Microscopy

Cells were fixed with PBS solution containing 4% paraformaldehyde for 15 minutes at room temperature and then permeabilized with a solution of DMEM containing 0.05% saponin. The slides were incubated for 1 hour with a primary antibody. After several washes, the plates were incubated for 1 hour in the dark in the presence of an anti-mouse or anti-rabbit Alexa Fluor 488 antibody (Invitrogen). Cells were then mounted on slides using a solution of Aqua-mount (Fisher Scientific, Ottawa, Canada) and observed using a confocal microscope (LSM510META).

### Western blotting

Cells were harvested, total soluble proteins were run on poly-acrylamide gels and transferred onto nitrocellulose membranes. The membranes were blotted for relevant proteins using specific primary antibodies (as described for each experiment). Secondary antibodies were horseradish peroxidase conjugated (GE Healthcare Life Sciences, Piscataway, NJ, USA) and detected with enhanced chemiluminescence detection reagent. Quantification of the digital images obtained was performed using ImageQuant 5.2 software (GE Healthcare Life Sciences).

### Membrane recruitment

MCF7 cells were transfected with GFP, ARF1 or ARF6, serum-starved overnight and stimulated with FBS (10%). Cells were then harvested in 200 μl of phosphate-buffered saline buffer containing protease inhibitors. Cell membranes were disrupted by passing three times through a 27G1/2 syringe. Cell lysates were then centrifuged for 10 minutes at 500 g to discard nuclei and cellular debris, and the supernatants were ultracentrifuged at 100,000 g (30 minutes, 4°C) to isolate cytosolic and membrane fractions. Membrane pellets were then lysed for 10 minutes in 100 μl of ice-cold TGH buffer. Samples were run on a 10% polyacrylamide gel. Proteins were detected by immunoblot analysis using specific antibodies.

### Wound healing assays

MCF7 cells were transfected separately with GFP, ARF1, ARF6 (48h) and seeded onto coverslips. Forty-eight hours post-transfection, confluent cells were serum-starved or not for 8 hours. Three scratches per well were then performed using a micropipette tip. Cells were treated with 1% or 10% FBS, and left for 16 hours or 5 days. Cells were live tracked or fixed and stained as in [8]. Image analysis was performed using Fiji (National Institutes of Health, USA) and Icy softwares (Institut Pasteur, Paris, France).

### Invasion assays

Cells were transfected with GFP, ARF1 or ARF6 (48 hours) and serum-starved overnight before the assay. Briefly, cells were trypsinized and seeded into Boyden chambers (24-well inserts with 8-μm pore, precoated with Matrigel), and one hour after plating, cells were stimulated with FBS. After 20 hours, cells were fixed using 4% paraformaldehyde and incubated with 0.1% crystal violet. The number of cells in the lower chamber was assessed as in [10].

### RNA extraction

Total RNA was extracted from MDA-MB-231 with TRIzol reagent (Life Technologies, Carlsbad, CA) according to the manufacturer's instructions. Real time-PCR was performed by the genomic platform at the IRIC's Genomics Core Facility (Université de Montréal, Montreal, Canada).

### Apoptosis analysis

Following transfection (24 hours), cells were treated for 48 hours with vehicle or etoposide (100 μM). Apoptosis induction was determined by Annexin V-FITC (Bd bioscience, 556419) and propidium iodide (PI) (MBL, Nakaku, Nagoya, JP) double staining, following the manufacturer's instructions. Briefly, 10^6^ cells were washed in PBS, resuspended in 500 μl of binding buffer (10 mM Hepes, pH 7.4, 150 mM NaCl, 5mM KCl, 1mM MgCl_2_, 1.8 mM CaCl_2_) and stained with 2 μl of Annexin V-FITC and 1 μl of PI (1mg/mL) for 10 minutes on ice. Analysis was performed using a FACS Canto (BD Biosciences, Bedford, MA, USA) flow cytometer and data were analyzed using the FlowJo software.

### Co-immunoprecipitation

MDA-MB-231 cells were kept in serum-free medium overnight and stimulated with EGF (100 ng/ml) for the indicated times. Co-immunoprecipita­tion experiments were conducted as previously described [50]. Briefly, cells were lysed into TGH buffer, and EFGR was immunoprecipitated using an anti-EGFR antibody. To ensure the specificity of the interaction, we have included a control condition where immunoprecipitations were performed in the absence of EGFR antibody. In­teracting, pan tyrosine was assessed by Western blot analysis.

### Statistical analysis

Statistical analyses were performed using Prism (GraphPad, (ver. 4.0a); San Diego, CA, USA). As indicated in the Figure legends, either a one-way or two-way analysis of variance followed by either a Tukey's, Bonferroni's or Dunnett's multiple comparison tests were used.

## SUPPLEMENTARY MATERIAL FIGURES


