# Supplementary material for: ADP-ribosylation factor 1 expression regulates epithelial-mesenchymal transition and predicts poor clinical outcome in triple-negative breast cancer
Source: Oncotarget. 2016 Feb 18;7(13):15811–27. doi: 10.18632/oncotarget.7515 (PMC4941279; doi:10.18632/oncotarget.7515)
Supplement: Supplementary file 1 [file oncotarget-07-15811-s001.pdf]

ADP-ribosylation factor 1 expression regulates epithelial-mesenchymal transition and predicts poor clinical outcome in triple-negative breast cancer

Supplementary Material

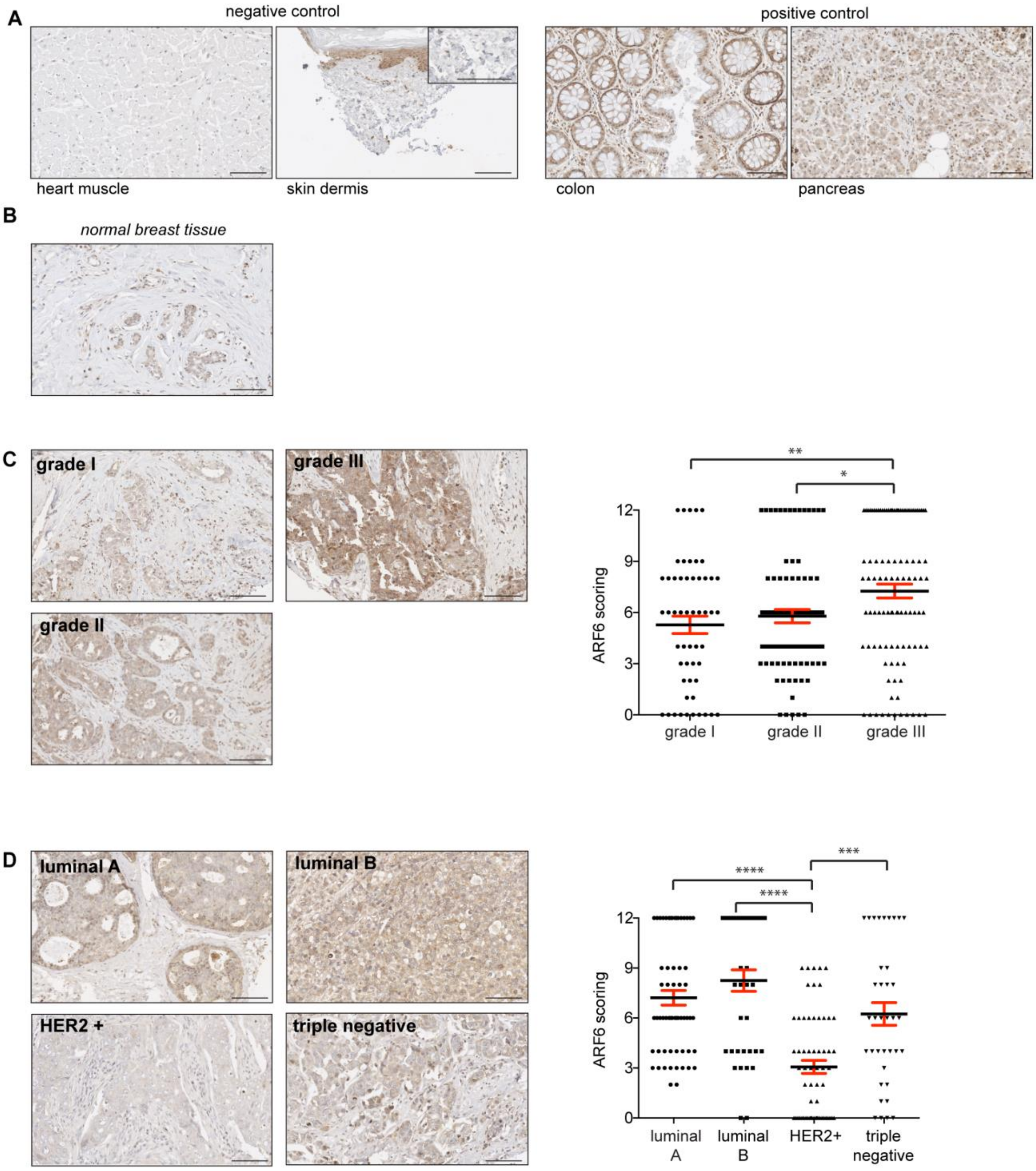

Supplemental Figure 1. ARF6 expression correlates with molecular subtypes of breast cancer and is associated with tumor grade. (A) Representative IHC labeling with ARF6 in normal human tissue. Heart muscle and skin dermis were chosen as negative controls, while colon and pancreas served as positive controls. Each sample was incubated with anti-ARF6 antibody. Scale bars, 100 µm. (B) Expression of ARF6 in normal breast tissues. Samples correspond to individual breast tissue from the same TMA and incubated with anti-ARF6 antibody. Scale bar, 100 µm. (C) ARF6 expression in breast cancers tissue samples according to histological grades. Samples are from the same TMA described as in A. Scale bars, 100 µm. Graph showing ARF6 labeling intensity in breast cancer tissue samples according to histological grade. Grade I n=54, II n=84 and III n=83, dataset including 231 patients. (D) Expression of ARF1 in different molecular subtypes of breast cancer. Samples correspond to individual breast cancer tissue from the same TMA and incubated with anti-ARF1 antibody. Scale bars, 100 µm. Graph depicting ARF1 labeling intensity of breast cancer tissue samples according to molecular subtype. Luminal A n=60, luminal B n=39, HER2+ n=61 and triple-negative n=35, dataset including 195 patients. In C and D, significance was measured by one-way ANOVA followed by Tukey's multiple comparison tests. * $p < 0.05$, ** $p < 0.01$, *** $p < 0.001$, ****$p < 0.0001$.

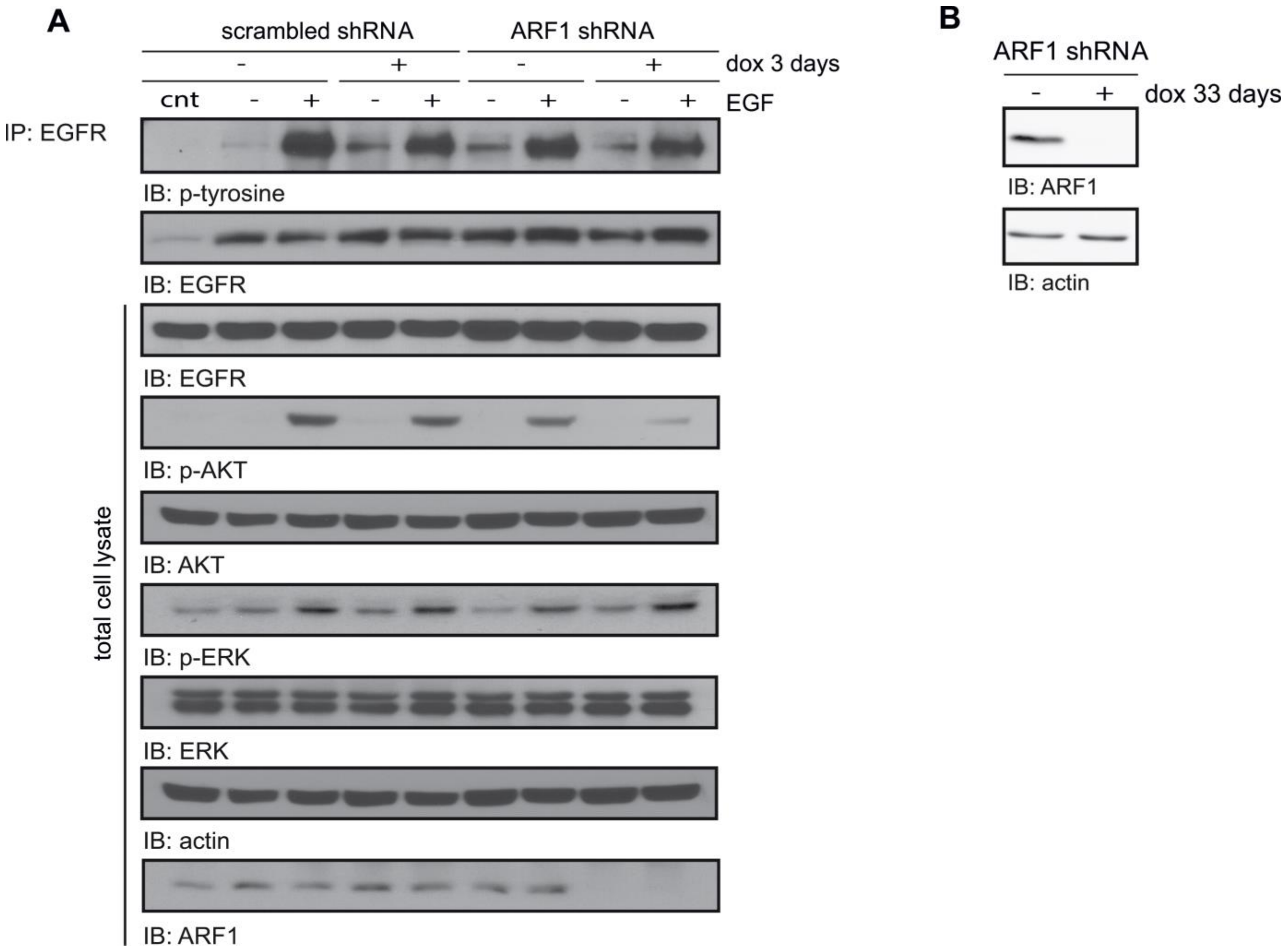

Supplemental Figure 2. Validation of ARF1 shRNA in MDA-MB-231 breast cancer cells. (A) Control (cnt; scrambled) or ARF1 knockdown MDA-MB-231 cells treated with +/- doxycycline for 3 days were stimulated with EGF (100 ng/ml) for 2 minutes. EGFR was immunoprecipitated and associated phospho-tyrosines were detected by Western blotting. Endogenously expressed ERK, AKT, actin and ARF1 were detected by Western blot. (B) ARF1 shRNA tansfected MDA-MB-231 cells were treated or not with doxycycline for 33 days. ARF1 and actin were detected as in (A). Data are representative of three experiments (A and B).

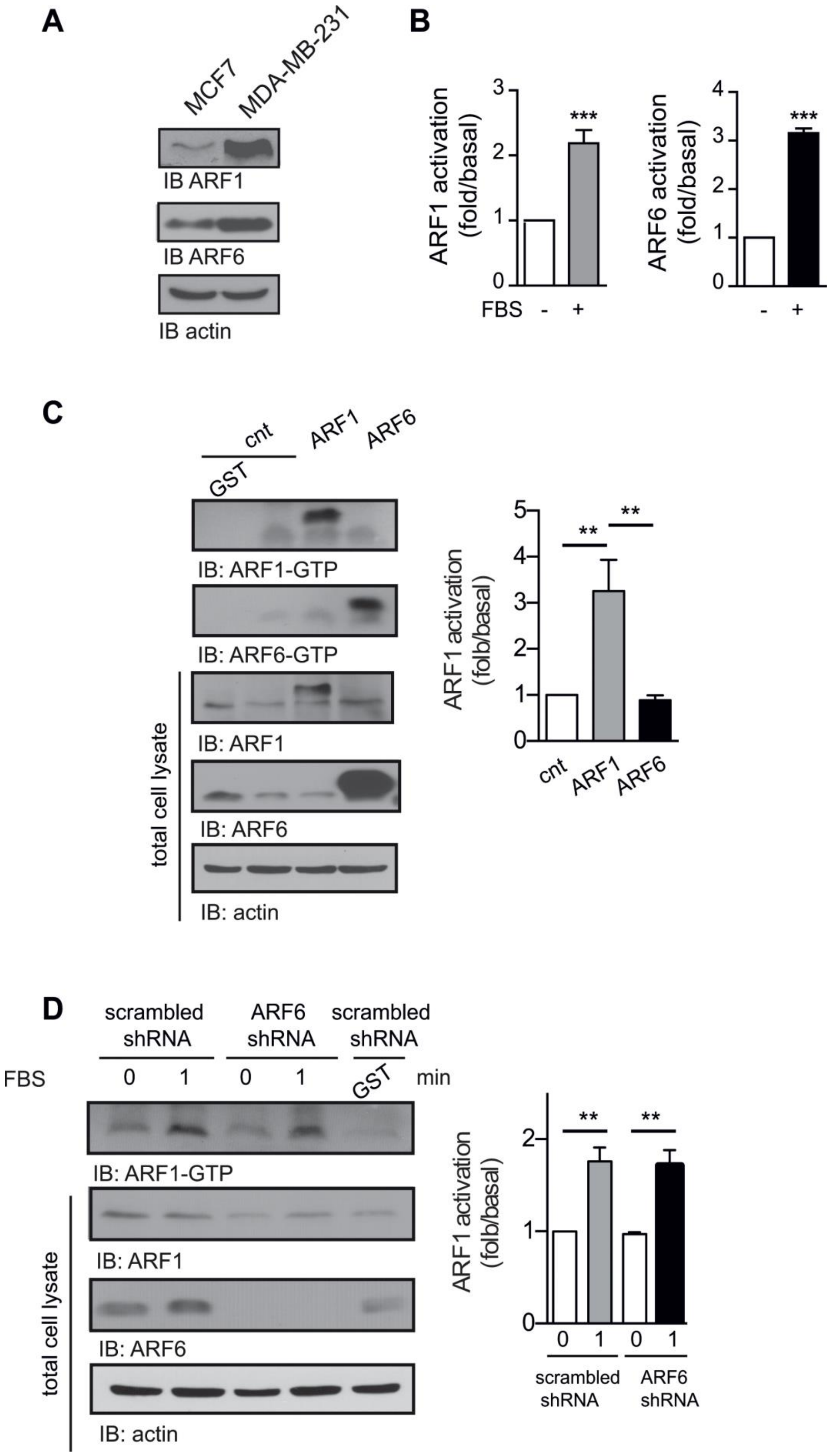
A
MCF7
MDA-MB-231
IB ARF1
IB ARF6
IB actin
B
ARF1 activation (fold/basal)
***
FBS - +
ARF6 activation (fold/basal)
***
- +
C
cnt
GST
ARF1
ARF6
IB: ARF1-GTP
IB: ARF6-GTP
IB: ARF1
IB: ARF6
IB: actin
total cell lysate
ARF1 activation (folb/basal)
**
**
cnt
ARF1
ARF6
D
scrambled shRNA
ARF6 shRNA
scrambled shRNA
FBS
0
1
0
1
GST
min
IB: ARF1-GTP
IB: ARF1
IB: ARF6
IB: actin
total cell lysate
ARF1 activation (folb/basal)
**
**
scrambled shRNA
ARF6 shRNA

Supplemental Figure 3. **ARF activation and localization in MCF7 cells.** (A) Endogenous ARF1, ARF6 and actin were detected by Western blotting. (B) Cells were stimulated with 10% FBS for 4 hours. Activated ARF1 or ARF6 was detected by GST pulldown assay. These experiments are representative of three others. *** $p < 0.001$ compared to unstimulated conditions. (C) MCF7 cells were transfected with empty vector, ARF1 or ARF6 and left in high serum media (10% FBS). Activated ARF1 or ARF6 was assessed as in A. Data are the mean ± SEM of three experiments. Statistical analyses were performed using a one-way ANOVA followed by a Bonferroni's multiple comparison tests. **$p<0.01$. (D) Activation of ARF1 was assessed as in A in cells transfected with either control (scrambled) or ARF6 shRNA. For each condition, cells were stimulated or not with 10% FBS for 1 minute. Activated ARF1 was assessed as in A. Data are the mean ± SEM of four experiments. Statistical analysis were calculated using a two-ways ANOVA followed by a Bonferroni's multiple comparison test. **$p<0.01$.

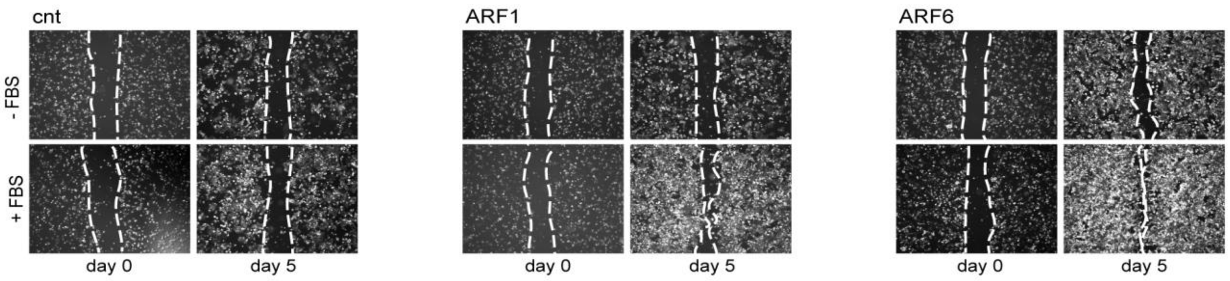

Supplemental Figure 4. **Overexpression of ARF controls proliferation of MCF7 cells.** Cells were transfected with an empty vector, ARF1 or ARF6, and wound healing assays were performed after 5 days in restricted medium or not. Images are representative of three other experiments performed in triplicate.

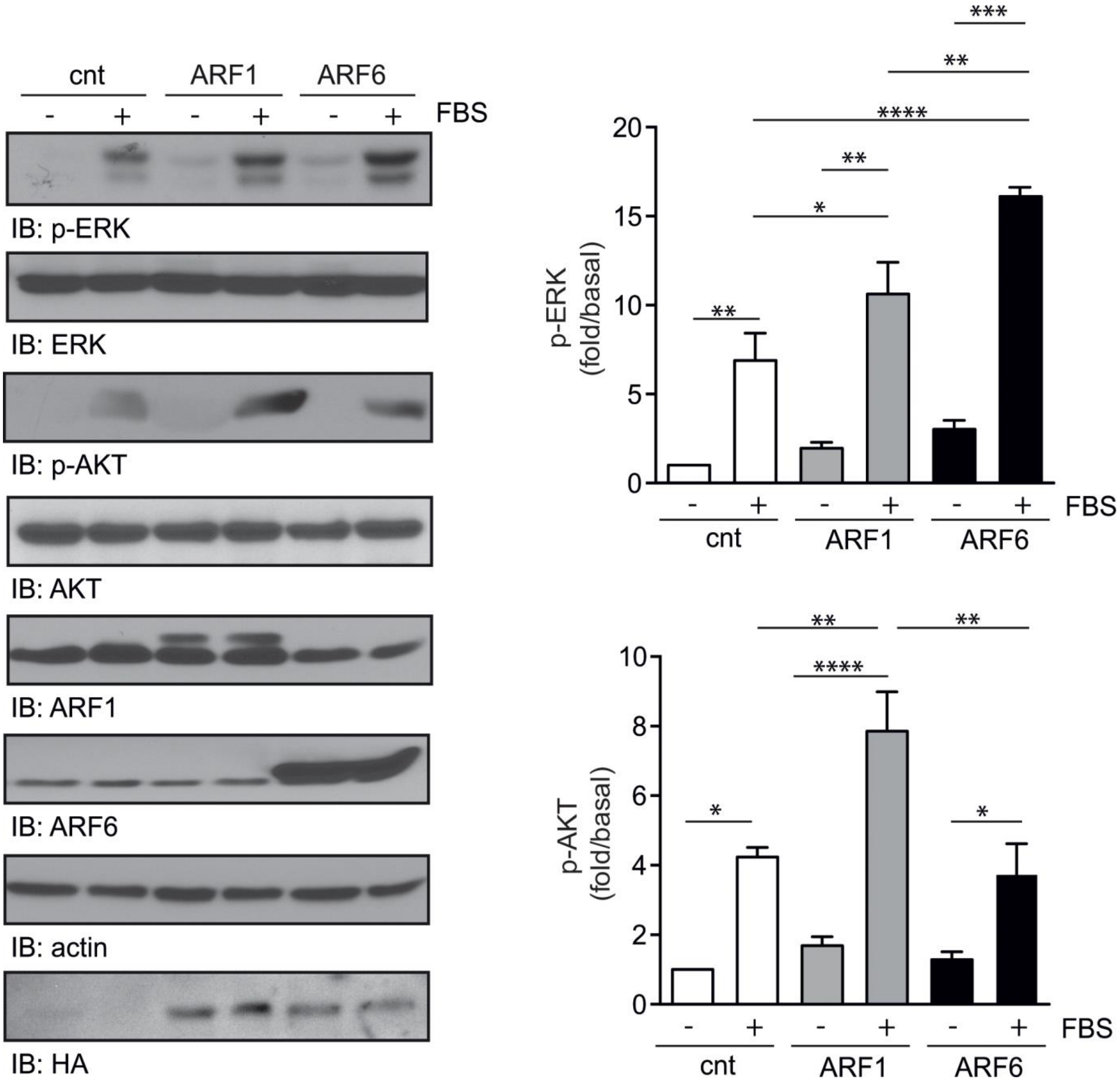

Supplemental Figure 5. **Overexpression of ARF in MCF7 cells potentiates activation of the MAPK and PI3K pathways.** MCF7 cells were transfected with empty vector, ARF1 or ARF6. Cells were stimulated with 10% FBS for 4 hours. Activation of ERK and AKT was assessed using phosphor-specific antibodies. Levels of ERK, AKT, ARF1, ARF6, actin and HA-tagged protein were detected by Western blotting. Data are the mean ± SEM of three experiments. Significance was determined by two-way ANOVA followed by Bonferroni's multiple comparison tests. *$p < 0.05$, **$p <0.01$, ***$p < 0.001$, ****$p < 0.0001$.
